# Ginsenoside Rb1 Alleviates Alcohol-Induced Liver Injury by Inhibiting Steatosis, Oxidative Stress, and Inflammation

**DOI:** 10.3389/fphar.2021.616409

**Published:** 2021-02-26

**Authors:** Yuqi Lai, Qinxiang Tan, Shu Xv, Sha Huang, Yuhua Wang, Yunjia Li, Ting Zeng, Chan Mo, Yuyao Chen, Shaohui Huang, Chuying Zhou, Lei Gao, Zhiping Lv

**Affiliations:** ^1^ School of Traditional Chinese Medicine, Southern Medical University, Guangzhou, China; ^2^ Renal Division, Beijing University of Chinese Medicine Shenzhen Hospital (Longgang), Shenzhen, China; ^3^ Oncology Department of Shenzhen Hospital of University of Chinese Academy of Sciences, Shenzhen, China; ^4^ The Key Laboratory of Molecular Biology, State Administration of Traditional Chinese Medicine, School of Traditional Chinese Medicine, Southern Medical University, Guangzhou, China

**Keywords:** ginsenoside Rb1, alcoholic liver disease, oxidative stress, inflammation, zebrafish

## Abstract

Alcoholic liver disease (ALD) has become a heavy burden on health worldwide. Ginsenoside Rb1 (GRb1), extracted from *Panax quinquefolium* L*.*, has protective effects on many diseases, but the effect and mechanisms of GRb1 on ALD remain unknown. This study aimed to investigate the protective effects of GRb1 on ALD and to discover the potential mechanisms. Zebrafish larvae were exposed to 350 mM ethanol for 32 h to establish a model of acute alcoholic liver injury, and the larvae were then treated with 6.25, 12.5, or 25 μM GRb1 for 48 h. The human hepatocyte cell line was stimulated by 100 mM ethanol and meanwhile incubated with 6.25, 12.5, and 25 μM GRb1 for 24 h. The lipid changes were detected by Oil Red O staining, Nile Red staining, and triglyceride determination. The antioxidant capacity was assessed by fluorescent probes *in vivo*, and the expression levels of inflammatory cytokines were detected by immunohistochemistry, immunofluorescence, and quantitative real-time PCR. The results showed that GRb1 alleviated lipid deposition in hepatocytes at an optimal concentration of 12.5 μM *in vivo*. GRb1 reversed the reactive oxygen species accumulation caused by alcohol consumption and partially restored the level of glutathione. Furthermore, GRb1 ameliorated liver inflammation by inhibiting neutrophil infiltration in the liver parenchyma and downregulating the expression of nuclear factor-kappa B pathway-associated proinflammatory cytokines, including tumor necrosis factor-α and interleukin-1β. This study revealed that GRb1 has a protective effect on alcohol-induced liver injury due to its resistance to lipid deposition as well as antioxidant and anti-inflammatory actions. These findings suggest that GRb1 may be a promising candidate against ALD.

## Introduction

Alcoholic liver disease (ALD) is induced by excessive alcohol consumption and has become the main liver disease worldwide. ALD includes alcoholic fatty liver, alcoholic steatohepatitis, fibrosis, and cirrhosis, and it can result in hepatocellular cancer (Seitz et al., 2018). Chronic alcohol consumption or binge drinking raises the blood alcohol concentration, increases exposure of the liver to alcohol, and activates alcohol metabolic enzymes, leading to the accumulation of acetaldehyde, an alcohol oxidative metabolite. Excessive acetaldehyde and ethanol levels in the liver cause DNA lesions, DNA adducts, and protein adducts, and they activate cytochrome p450 family 2 subfamily E member 1 to generate a large number of reactive oxygen species (ROS), resulting in oxidative stress, inflammation, and cell injury (Setshedi et al., 2010). In brief, the pathogenesis of ALD is related to acetaldehyde-mediated toxicity, oxidative stress, and lipid peroxidation as well as cytokine and chemokine-induced inflammation (Das and Vasudevan, 2007; Wu and Cederbaum, 2009; Dunn and Shah, 2016). In the past, numerous studies on ALD have used chronic models of long-term alcohol feeding, but less attention has been given to liver injury due to acute alcohol exposure. In addition, a few therapies are now being used as therapeutic guidelines for ALD, including alcohol withdrawal, corticosteroids, pentoxifylline usage to block the transcription of tumor necrosis factor-α (TNF-α), and liver transplantation (Ohashi et al., 2018; Kong et al., 2019). New treatment strategies need to be identified and verified to treat ALD. Recently, the application of natural agents in ALD treatment has attracted much attention (Kim et al., 2016).

Ginsenosides are the main bioactive compounds in ginseng roots and have antioxidant, anti-inflammatory, and lipid-decreasing effects (Kitts et al., 2000; López et al., 2007; Kim et al., 2017). Ginsenoside Rb1 (GRb1), the most abundant ginsenoside in *Panax quinquefolium* L*.* (Lim et al., 2005), has been proven to alleviate acute lung injury, myocardial infarction, colitis, osteoarthritis, and cerebral ischemic reperfusion injury (Li et al., 2014; Chen et al., 2016; Zheng et al., 2017; Liu et al., 2018; Shaukat et al., 2019). However, the role and molecular mechanism of GRb1 in ALD have not been reported. Consequently, the GRb1 pharmacological agent in ALD needs to be explored and elucidated to provide scientific evidence for its clinical application.

In the past 20 years, due to the high degree of genetic similarity to humans and the high conservation of liver cell types, zebrafish have become a superior laboratory model with the attraction of large spawning, rapid development, and transparent young fish (Goessling and Sadler, 2015; Cassar et al., 2020). Additionally, zebrafish have become more effective tools for basic research and drug discovery as several methods have been developed and applied to zebrafish to alter gene transcription and function (Cornet et al., 2018). Thus, zebrafish are an appropriate model to explore the therapeutic effects of natural drugs on ALD based on high-throughput ability.

## Materials and Methods

### Antibodies and Reagents

**FIGURE 1:**
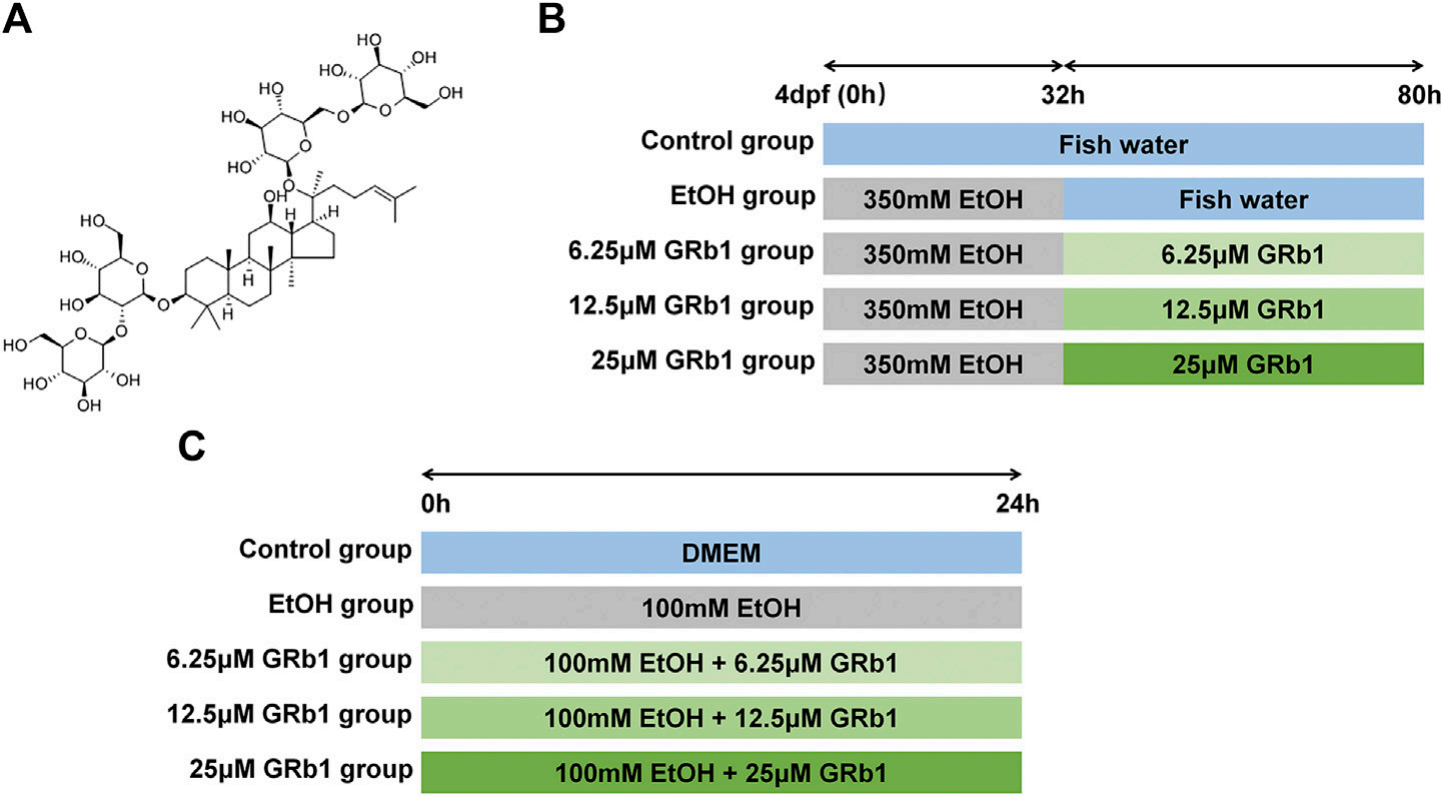
Study design. **(A)** The chemical structure of GRb1. **(B)** Experimental process in zebrafish larvae. **(C)** Experimental procedure in L-O2 cells.

The GRb1 analytical standard (CAS No: 41753-43-9; C54H92O23) with ≥98.0% purity (HPLC) was purchased from Chengdu Must Biotechnology Co., Ltd. (Chengdu, China). The chemical structure of GRb1 is presented in Figure 1A. Antibodies against NF-κB (ET1603-12) were obtained from HuaAn Biotechnology Co., Ltd. (Hangzhou, China), and anti-TNF-α (ab1793) antibodies were acquired from Abcam (Cambridge, United Kingdom). Horseradish peroxidase-labeled goat anti-mouse/rabbit IgG (GK500710) was procured from Gene Tech (Shanghai, China). Alexa Fluor594-conjugated goat anti-rabbit IgG (#8889) and Alexa Fluor594-conjugated goat anti-mouse IgG (#8890) were obtained from Cell Signaling Technology (United States).

### Zebrafish Experiment

The zebrafish strain used in this study were the wild-type, the hepatocyte-specific enhanced green fluorescent protein (EGFP) expression line [Tg (lfabp10α: EGFP)] and the transgenic line with neutrophil-specific EGFP expression [Tg (MPO: EGFP)]. Adults and larvae were raised at 28.5°C and with a 14 h light/10 h dark period. Egg water (0.5 mg/L methylene blue) was provided for embryos. All zebrafish maintenance and experiments were approved by the Institutional Animal Care and Use Committee of Southern Medical University.

Zebrafish larvae at 4 days post fertilization were divided into the following five groups at random: the control group was raised in fish water; the ethanol group was exposed to 350 mM ethanol for 32 h and then transferred to fish water; three groups of GRb1 treatment were treated with 350 mM alcohol for 32 h followed by the intervention of 6.25, 12.5, or 25 μM GRb1 for 48 h. The experimental process is shown in Figure 1B. After the treatment, larvae were fixed in 4% paraformaldehyde (for paraffin and frozen sections).

### Cell Treatment

Human fetal hepatocyte cell line (L-O2) was obtained from the Key Laboratory of Molecular Biology, School of Traditional Chinese Medicine of Southern Medical University. Cells were cultured in Dulbecco’s Modified Eagle’s Medium (DMEM) containing 10% fetal bovine serum, 100 IU/ml penicillin, and 100 mg/ml streptomycin (Gibco, Carlsbad, CA, United States) in a humidified atmosphere with 5% CO_2_ at 37°C. Cells were stochastically separated into the following five groups: the control group, the model group, and three GRb1 treatment groups. An *in vitro* ALD model was established by using 100 mM ethanol to stimulate cells for 24 h. Cells in three GRb1 intervention groups were incubated with 100 mM ethanol and GRb1 (6.25, 12.5, and 25 μM) for 24 h. The procedure is shown in Figure 1C. Cells were fixed in 4% paraformaldehyde or lyzed 24 h later.

### Hematoxylin and Eosin Staining

After being fixed in 4% paraformaldehyde at 4°C overnight, zebrafish larvae were embedded in paraffin and cut into 4 μM sections. Specimens were dewaxed, rehydrated, stained with H&E, dehydrated, cleared, and sealed for routine histology. Finally, H&E sections were captured by a Nikon Eclipse Ni-U light microscope (Nikon, Tokyo, Japan).

### Whole-Mount Staining of Oil Red O

Fixed zebrafish larvae were washed with phosphate-buffered saline (PBS) and then permeated successively with 20, 40, 80, and 100% 1,2-propylene glycol (Sigma, United States) for 15 min each. Larvae were then stained with 0.5% Oil Red O (Sigma, United States) for 1 h at 65°C in the dark. The samples were then incubated in 100% 1,2-propylene glycol for 60 min and rinsed with 80, 40, and 20% 1,2-propylene glycol for approximately 30 min. Finally, larvae were imaged by an Olympus U-HGLGPS microscope (Tokyo, Japan) under a bright field.

### Oil Red O Staining of Cryosections and Cells

Fixed zebrafish larvae were dehydrated in 30% sucrose at 4°C for 3 days, embedded in optimal cutting tissue (OCT) compound (Leica, Germany), and sliced into 14 μM sections. Cryosections were washed with water to remove OCT, incubated in 100% 1,2-propylene glycol for 5 min, and stained with 0.7% Oil Red O at 60°C for 10 min in the dark. Excess dye was rinsed off with 85% 1,2-propylene glycol and PBS to keep the background clean. Sections were imaged using a Nikon Eclipse Ni-U light microscope (Nikon, Tokyo, Japan).

The Oil Red O staining procedure for cells was the same as that of cryosections. The fixed cells were washed with PBS, incubated with 100% 1,2-propylene glycol, stained with 0.7% Oil Red O, and decontaminated in 85% 1,2-propylene glycol and PBS. The staining results were imaged using a Nikon Eclipse Ni-U optical microscope (Nikon, Tokyo, Japan).

### Nile Red Staining

Before staining with Nile Red dye (Sigma, United States), zebrafish larvae cryosections were washed with water to remove OCT, while fixed cells were washed with PBS. Both cells and zebrafish slides were then incubated with Nile Red solution for 10 min (at a concentration of 0.5 μg Nile Red dissolved in 1 ml of acetone), washed with PBS, and stained with DAPI (Solarbio Life Science, China) for 5 min protected from light. Samples were imaged using a Nikon Eclipse Ni-U fluorescence microscope (Nikon, Tokyo, Japan).

### Triglyceride Detection

Triglycerides in cells were measured using the Triglyceride Assay Kit (Nanjing Jiancheng Bioengineering Institute, China) following the manufacturer’s instructions and were normalized to the total protein concentration as determined by the BCA Protein Assay Kit (Beyotime, Shanghai, China).

### Superoxide and Glutathione Probe Detection

Dihydroethidium (DHE, Beyotime, Shanghai, China) for ROS detection and a naphthylamide-sulfoxide-based fluorogenic probe (Na-8, Future-Chase Biotechnology Co., Ltd.; FYRK-FP-01-003KY) for GSH determination were used in zebrafish larvae. After treatment, the live larvae were incubated with a 10 μM solution for 10 min at 28°C in the dark. The larvae were then moved to fish water and narcotized with 0.2% tricaine (Sigma, United States). The distribution and intensity of DHE and NA-8 fluorescence were visualized with a Nikon Eclipse Ni-U fluorescence microscope (Nikon, Tokyo, Japan).

The ROS probe (Beyotime; S0033) and Na-8 probe were used in L-O2 cells. Cells were seeded in 96-well plates (1 × 10^4^ cells/well) for 24 h and then subjected to the above treatments. Finally, the cells were incubated with the ROS probe (10 μM) or Na-8 probe (10 μM), respectively, for 30 min in the dark. The cellular fluorescence intensity was collected by a fluorescence microplate reader (BioTek Epoch, America) with excitation and emission wavelengths of 488 nm and 525 nm for the ROS probe and 350 nm and 450 nm for the Na-8 probe. Cells were photographed under a fluorescence microscope (Nikon, Tokyo, Japan).

### Malondialdehyde Detection

Cells were lysed in RIPA cell lysis buffer (Beyotime, Shanghai, China), and the supernatant was collected to detect MDA levels by applying an MDA Assay Kit (Beyotime, Shanghai, China) according to the manufacturers’ instructions. MDA levels were normalized to the total protein concentration as determined by the BCA Protein Assay Kit.

### GSH Detection

Before determination, different groups of cells were counted. The GSH levels in cells were detected by applying a GSH and GSSG Assay Kit (Beyotime, Shanghai, China) according to the manufacturers’ protocol and were normalized to the number of cells.

### Immunochemistry Assay

After deparaffinization in xylene and rehydration in descending ethanol concentrations, paraffin sections were boiled in sodium citrate for 10 min to repair antigen and incubated with 3% H_2_O_2_ in methanol for 10 min to inactivate endogenous peroxidase enzyme in tissues after cooling to room temperature. The sections were washed with PBS, sealed with blocking buffer (5% normal goat serum and 0.1% Triton-X in PBS) for 2 h at room temperature, and incubated with rabbit anti-NF-κB antibody (1:200 dilution) or mouse anti-TNF-α antibody (1:150 dilution) at 4°C overnight. On the second day, samples were rinsed with PBS and then incubated in a horseradish peroxidase-labeled goat anti-mouse/rabbit secondary antibody for 1 h at room temperature. Slides were then washed with PBS, stained with DAB for 10 min, and counterstained with hematoxylin. Finally, the samples were dehydrated, cleared, and sealed with neutral gum. The stained sections were observed and imaged with a Nikon Eclipse Ni-U optical microscope (Nikon, Tokyo, Japan).

### Immunofluorescence Assay

Fixed cells were washed with PBS, sealed in flesh blocking buffer (PBS containing 5% normal goat serum and 0.1% Triton-X) for 2 h at room temperature, and incubated with a rabbit anti-NF-κB antibody (1:200 dilution) or mouse anti-TNF-α antibody (1:150 dilution) at 4°C overnight. On the second day, cells were rinsed with PBS and incubated with Alexa Fluor594-conjugated goat anti-rabbit IgG or goat anti-mouse IgG secondary antibody (1:1,000 dilution) for 2 h at room temperature. After being washed with PBS, the samples were stained with DAPI for 5 min in the dark. The results were recorded by a Nikon Eclipse Ni-U fluorescence microscope (Nikon, Tokyo, Japan).

### Quantitative Real-Time PCR

Total RNA was extracted from 10 larvae using TRIzol reagent (15596018, Ambion™) following the manufacture’s protocol. RNA was reverse-transcribed into cDNA using a commercial kit (Evo M-MLV RT Kit with gDNA Clean for qPCR II, Accurate Biotechnology (Hunan) Co., Ltd.) according to the manufacturer’s protocol. qPCR was performed using a SYBR Green Premix Pro Taq HS qPCR Kit (Accurate Biotechnology (Hunan) Co., Ltd.) on a StepOnePlus™ System (Applied Biosystems). The reaction parameters of qPCR were as follows: predenaturation for 30 s at 95°C; 40 cycles of denaturation for 5 s at 95°C; and annealing and extending for 30 s at 60°C. The primers for each gene were purchased from BGI Tech Solutions (Beijing Liuhe) Co., Ltd. and are listed in Table 1. The β-actin gene was used as a reference, and the relative change was normalized to β-actin mRNA using the following formula: 2^−ΔΔCt^.

**TABLE 1:** Primers used to quantify mRNA levels.

Gene	FP sequence (5′-3′)	RP sequence (5′-3′)
β-Actin	ATG​GAT​GAG​GAA​ATC​GCT​GCC	CTC​CCT​GAT​GTC​TGG​GTC​GTC
TNF-α	ACC​AGG​CCT​TTT​CTT​CAG​GT	TGC​CCA​GTC​TGT​CTC​CTT​CT
IL-1β	TGG​ACT​TCG​CAG​CAC​AAA​ATG	CAC​TTC​ACG​CTC​TTG​GAT​GA

### Statistical Analysis

Statistical analyses were performed by one-way ANOVA followed by Tukey’s multiple comparison test, using SPSS 20.0. Data are represented as the means ± SEM. Statistical significance was recognized at a *p* value of less than 0.05. Graphs were created using GraphPad Prism version 5.01 software.

## Results

### GRb1 Alleviates Acute Alcohol-Induced Hepatic Steatosis in Zebrafish

**FIGURE 2:**
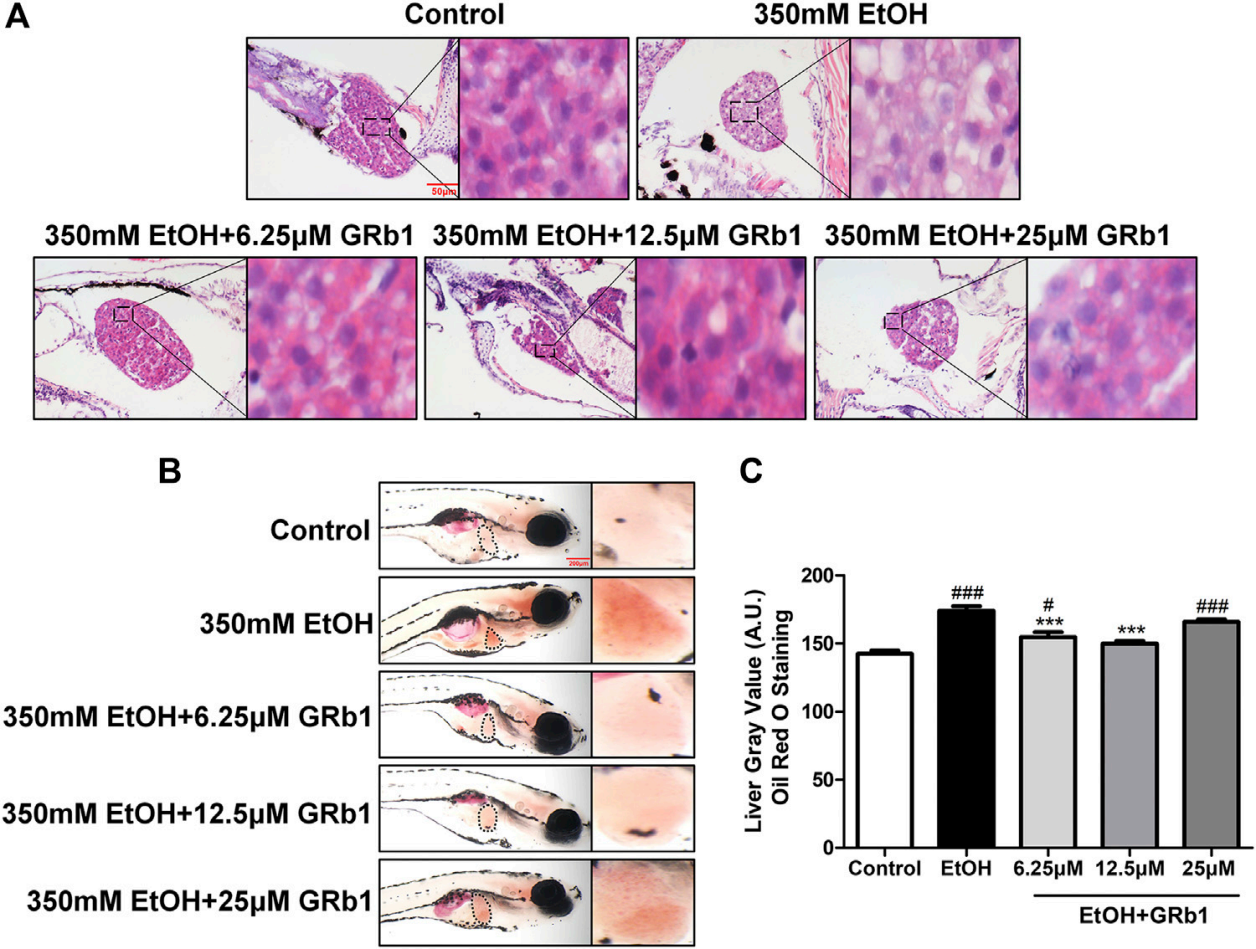
GRb1 alleviates alcohol-induced hepatic steatosis in zebrafish larvae. **(A)** H&E staining of zebrafish larvae. Figures are magnified as 400ⅹ. **(B)** Whole-mount staining with Oil Red O. Figures are magnified as 50ⅹ. **(C)** Quantitative analysis of the liver gray value of Oil Red O using ImageJ software. Data are shown as the mean ± SEM (*n* = 10 per group from two experiments). *p* < 0.05 (#), *p* < 0.01 (##), and *p* < 0.001 (###) compared with the control group; *p* < 0.05 (*), *p* < 0.01 (**), and *p* < 0.001 (***) compared with the 350 mM EtOH group.

**FIGURE 3:**
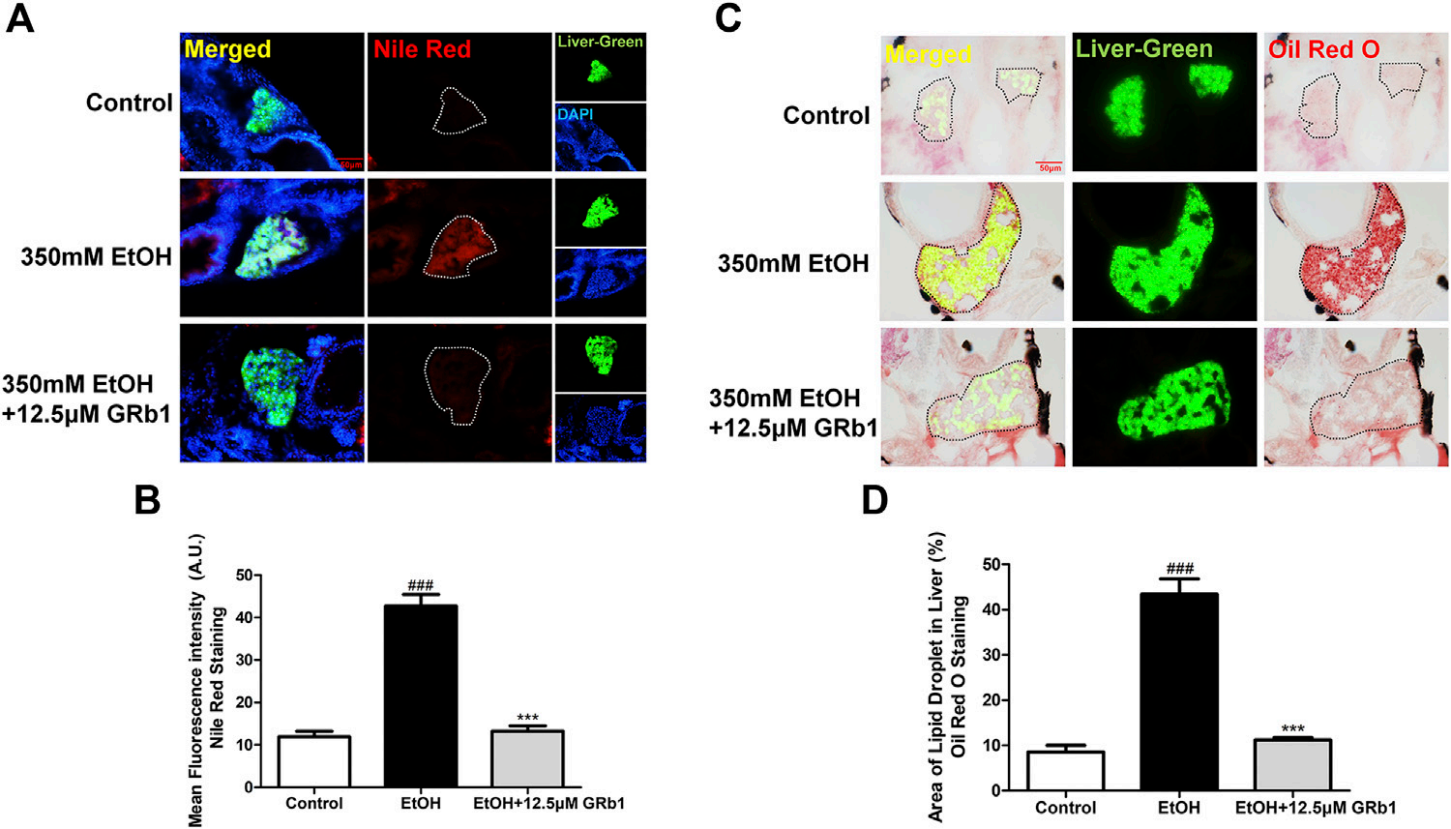
GRb1 significantly ameliorates hepatic steatosis caused by alcohol. **(A)** Nile Red staining of frozen liver sections from zebrafish larvae with liver-specific EGFP expression. Figures are magnified as 400ⅹ. **(B)** Quantitative analysis of the mean fluorescence intensity of Nile Red using ImageJ software. **(C)** Oil Red O staining of cryosections from zebrafish larvae. Figures are magnified as 400ⅹ. **(D)** Quantitative analysis of the area of lipid droplets in the liver based on Oil Red O staining using ImageJ software. Data are expressed as the mean ± SEM (*n* = 8–10 per group). *p* < 0.05 (#), *p* < 0.01 (##), and *p* < 0.001 (###) compared the control group; *p* < 0.05 (*), *p* < 0.01 (**), and *p* < 0.001 (***) compared with the 350 mM EtOH group.

Previous studies have shown that zebrafish larvae continuously stimulated with 350 mM ethanol for 32 h exhibit behavioral abnormalities, hepatomegaly, and steatosis of parenchymal hepatic cells (Passeri et al., 2009). Therefore, we exposed zebrafish larvae of 4 days post fertilization to 350 mM ethanol for 32 h to establish an acute alcoholic liver injury model. Based on this model, the protective effects of GRb1 on ALD were evaluated. H&E, Nile Red, and Oil Red O staining were applied to assess the fat accumulation level in hepatocytes, which is the earliest reaction of the liver to alcohol addiction. The H&E staining results showed that zebrafish larvae exposed to alcohol had severe lipid vacuoles within hepatocytes, while GRb1-treated groups had less vacuolization of the liver parenchyma (Figure 2A). The results of the whole-mount staining of Oil Red O agreed with H&E staining, which showed that the area and the number of lipid drops in the liver were significantly decreased after the treatment of GRb1 compared to those in the model group (Figures 2B,C). Although the results showed that 25μM GRb1 did not alleviate lipid deposition induced by alcohol, toxicological results (Supplementary Figure S1) indicated that 25μM GRb1 had no effect on survival rate, heart rate and body length of zebrafish. In other words, 25μM GRb1 had no toxic effect on zebrafish. Based on the pathological results and Oil Red O staining, we found that 12.5 μM was the optimum concentration of GRb1 to ameliorate hepatic steatosis. Based on the above result, we selected the 12.5 μM GRb1 group for further verification and intensive study. Nile Red staining (Figures 3A,B) and Oil Red O (Figures 3C,D) staining in cryosections provided further scientific evidence to confirm that 12.5 μM GRb1 effectively alleviated lipid deposition in the liver after ethanol exposure. These data revealed that GRb1 prevents hepatocytes from fatty degeneration induced by acute alcohol consumption.

### GRb1 Lowers Lipid Accumulation Caused by Alcohol in L-O2 Cells

**FIGURE 4:**
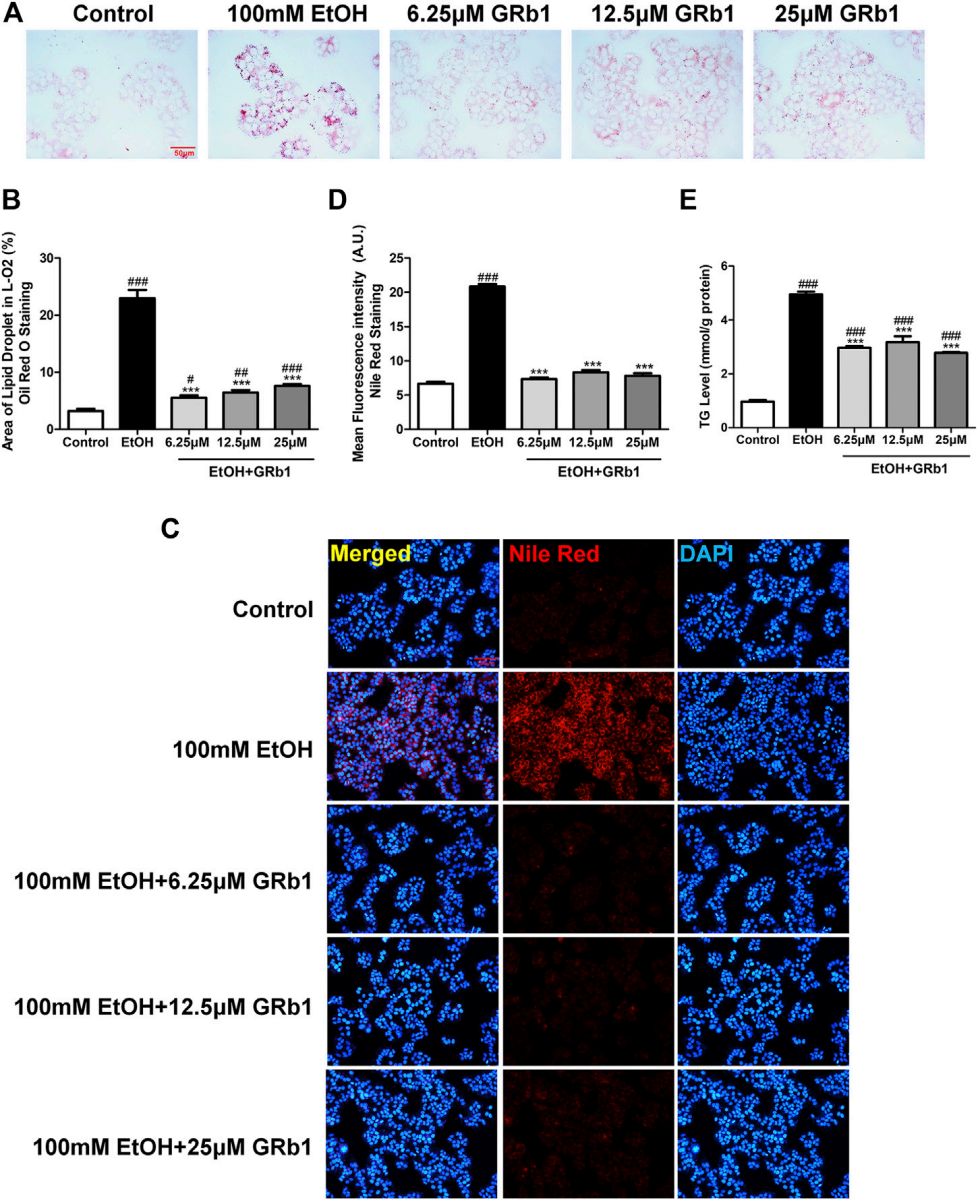
GRb1 reduces lipid accumulation stimulated by alcohol in L-O2 cells. **(A)** Oil Red O staining of L-O2 cells. Figures are magnified as 400ⅹ. **(B)** Quantitative analysis of the area of lipid droplets in L-O2 cells based on Oil Red O staining using ImageJ software. **(C)** Nile Red staining of L-O2 cells. Figures are magnified as 200ⅹ. **(D)** Quantitative analysis of the mean fluorescence intensity of Nile Red using ImageJ software. **(E)** Triglyceride levels in L-O2 cells. The data are displayed as the means ± SEM. *p* < 0.05 (#), *p* < 0.01 (##), and *p* < 0.001 (###) compared the control group; *p* < 0.05 (*), *p* < 0.01 (**), and *p* < 0.001 (***) compared with the 100 mM EtOH group.

We also verified the effect of GRb1 on lipid deposition *in vitro*. A considerable body of research has reported that exposing L-O2 cells to 100 mM ethanol for 24 h establishes an *in vitro* model of alcohol-induced liver damage (Lu et al., 2017; Jin et al., 2018). Therefore, this *in vitro* model was used to investigate the protective effect of GRb1 on fatty degeneration of hepatocytes. Consistent with the above results, both Oil Red O (Figures 4A,B) staining and Nile Red staining (Figures 4C,D) indicated that 100 mM ethanol stimulation for 24 h significantly induced intracellular lipid accumulation, but it was eliminated by GRb1. Triglyceride accumulation contributes greatly to steatosis. We found that intracellular triglyceride levels increased nearly 5 times with alcohol stimulation but decreased by nearly half in the GRb1 treatment group compared with the model group (Figure 4E). These results demonstrated that GRb1 is equally effective in reducing lipid accumulation induced by alcohol *in vitro*.

### GRb1 Protects Zebrafish Larvae and L-O2 Cells Against Oxidative Stress After Alcohol Exposure

**FIGURE 5:**
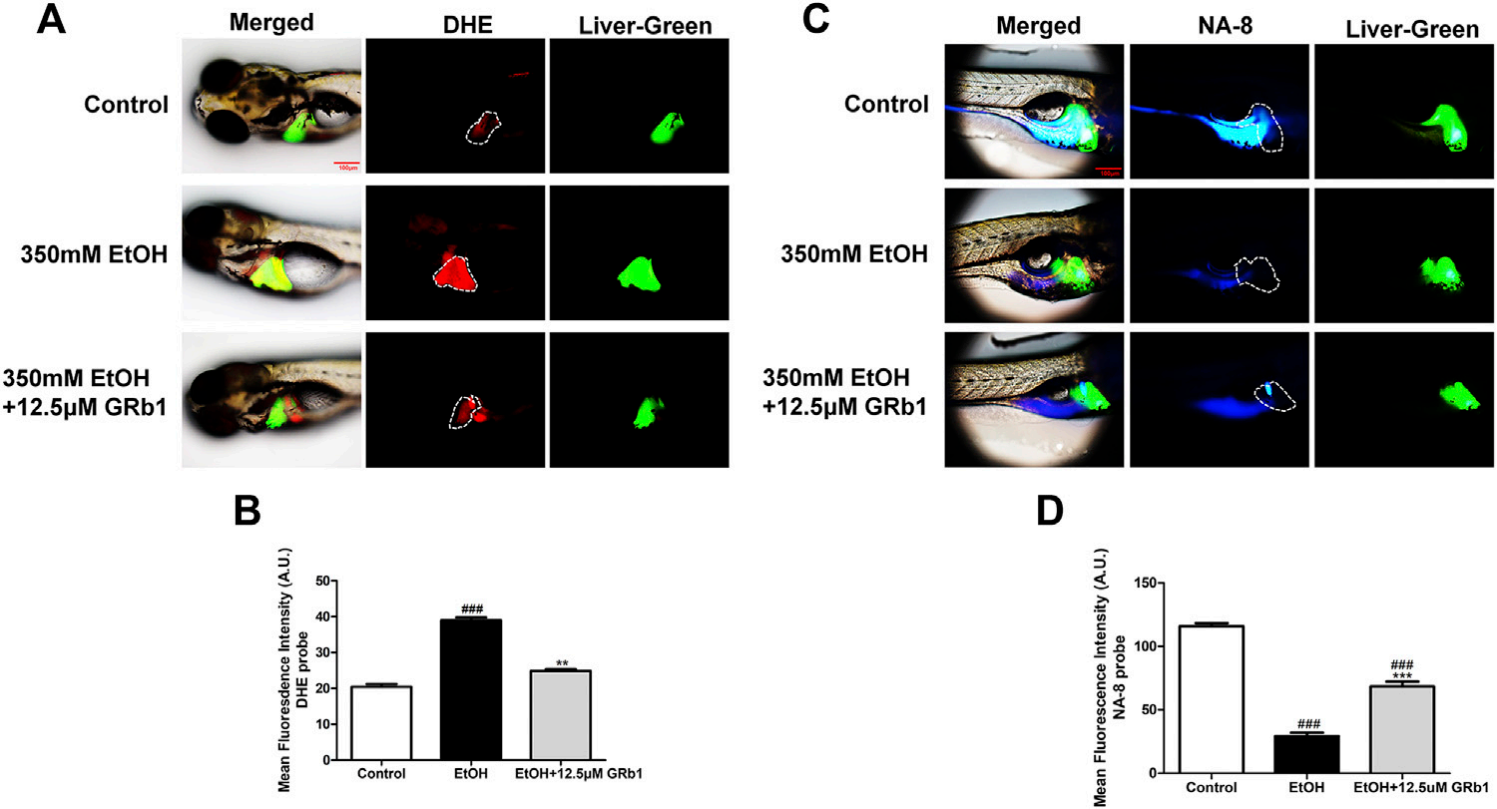
GRb1 protects zebrafish larvae against oxidative stress induced by alcohol exposure. **(A)** Fluorescence micrographs of DHE. Figures are magnified as 100ⅹ. **(B)** ROS quantification according to the mean intensity of red fluorescence using ImageJ software (n = 6–8 per group). **(C)** Fluorescence micrographs of NA-8. Figures are magnified as 100ⅹ. **(D)** GSH quantification according to the mean intensity of blue fluorescence using ImageJ software (*n* = 6–8 per group). Data are presented as the mean ± SEM. *p* < 0.05 (#), *p* < 0.01 (##), and *p* < 0.001 (###) compared the control group; *p* < 0.05 (*), *p* < 0.01 (**), and *p* < 0.001 (***) compared with the 350 mM EtOH group.

**FIGURE 6:**
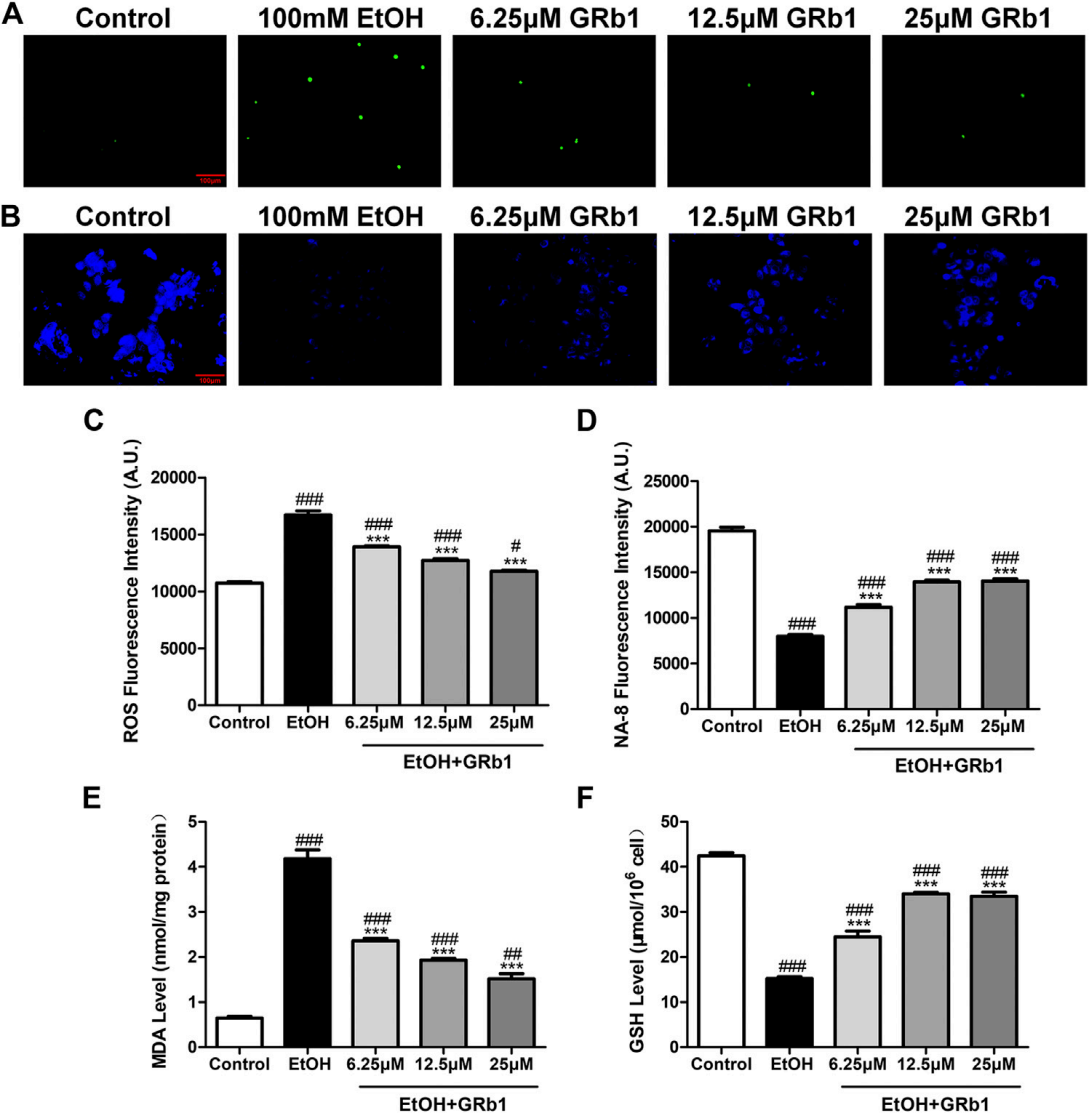
GRB1 reduces the oxidative stress caused by alcohol in L-O2 cells. **(A)** Fluorescence micrographs of ROS in L-O2 cells. Figures are magnified as 200ⅹ. **(B)** Fluorescence micrographs of NA-8 in L-O2 cells. Figures are magnified as 200ⅹ. **(C)** Cellular fluorescence intensity of ROS as measured using a fluorescence microplate reader. **(D)** Cellular fluorescence intensity of GSH as measured using a fluorescence microplate reader. **(E)** MDA levels in L-O2 cells. **(F)** GSH levels in L-O2 cells. The data are displayed as the means ± SEM. *p* < 0.05 (#), *p* < 0.01 (##), and *p* < 0.001 (###) compared the control group; *p* < 0.05 (*), *p* < 0.01 (**), and *p* < 0.001 (***) compared with the 100 mM EtOH group.

ROS play a preeminent role in the clinical and pathological spectrum of ALD (Ceni et al., 2014). ROS are toxic to cells, causing DNA damage, lipid peroxidation, and even cell death (Cederbaum et al., 2009). Mitochondrial GSH deficiency has been regarded as a contributor to ALD development as GSH functions in pathways responsible for ROS detoxification (Mantena et al., 2008). Therefore, DHE and NA-8 fluorescence probes, which track the distribution and levels of ROS and GSH, were used to evaluate ethanol-induced oxidative stress injury in the livers of zebrafish larvae and the protectiveness ability of GRb1. The results revealed that 12.5 μM GRb1 reversed ROS accumulation induced by alcohol consumption as indicated by intense red fluorescence appearing after alcohol exposure, and the fluorescence intensity decreased after treatment with 12.5 μM GRb1 (Figures 5A,B). *In vitro* experiments also confirmed this effect. The ROS probe in L-O2 cells showed higher ROS levels in the model group and decreased ROS levels in the GRb1 groups (Figures 6A,C). MDA detection in cells also supported this conclusion (Figure 6E).

In addition, the increased glutathione level in the livers of larvae after GRb1 administration also strongly supports the protective role of GRb1 against oxidative stress. The blue fluorescence emitted by the NA-8 probe weakened due to GSH depletion caused by ethanol. However, glutathione increased in the liver after intervention with 12.5 μM GRb1 (Figures 5C,D). The NA-8 probe in L-O2 cells showed the lowest GSH levels after alcohol stimulation and increased GSH levels in the GRb1 groups (Figures 6B,D). The GSH detection data in cells were consistent with the above results (Figure 6F). These outcomes suggested that GRb1 ameliorates ethanol-induced oxidative stress injury.

### GRb1 Ameliorates Liver Inflammation During Acute Alcoholic Injury, Including Neutrophil Infiltration and Proinflammatory Cytokines

**FIGURE 7:**
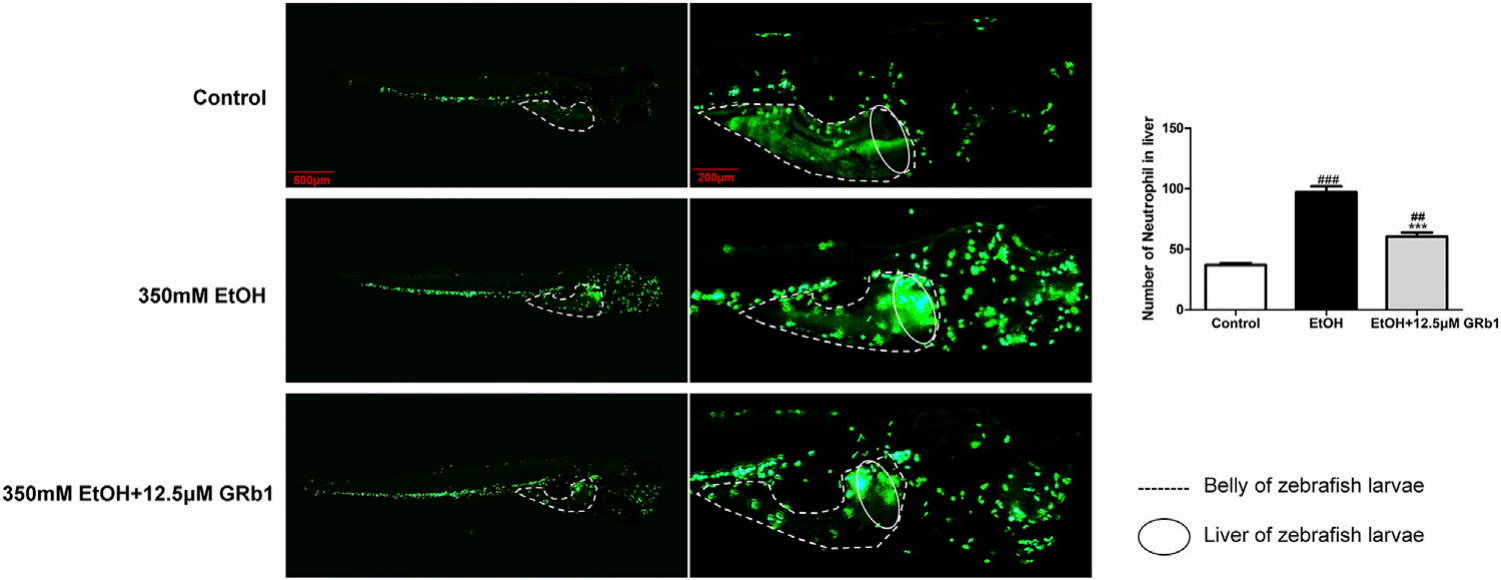
GRb1 alleviates neutrophil infiltration of the liver during acute alcohol injury. Neutrophil fluorescence micrographs and quantification of the number of neutrophils in the liver (within the cycle). After alcohol modeling and GRb1 administration, zebrafish larvae with neutrophil-specific EGFP expression were observed and imaged using an Olympus U-HGLGPS fluorescence microscope (Tokyo, Japan). Figures are magnified as 20 and 50ⅹ. The number of neutrophils in the liver was counted using particle analysis in ImageJ software. Data are presented as the mean ± SEM (*n* = 6–8 per group). *p* < 0.05 (#), *p* < 0.01 (##), and *p* < 0.001 (###) compared the control group; *p* < 0.05 (*), *p* < 0.01 (**), and *p* < 0.001 (***) compared with the 350 mM EtOH group.

Infiltration of the liver parenchyma by neutrophils is a striking feature of alcoholic steatohepatitis and plays pivotal roles in the development and progression of ALD (Jaeschke, 2002; Gao and Bataller, 2011). Studies on liver injury have indicated that neutrophils migrating to the liver parenchyma kill hepatocytes by releasing ROS and proteases (Ramaiah and Jaeschke, 2007a), possibly causing alcoholic liver damage. In our study, zebrafish with neutrophil-specific EGFP expression allowed instant observations. We found that alcohol exposure led to neutrophil infiltration in the liver. However, 12.5 μM GRb1 alleviated the migration of neutrophils into the liver parenchyma (Figure 7).

**FIGURE 8:**
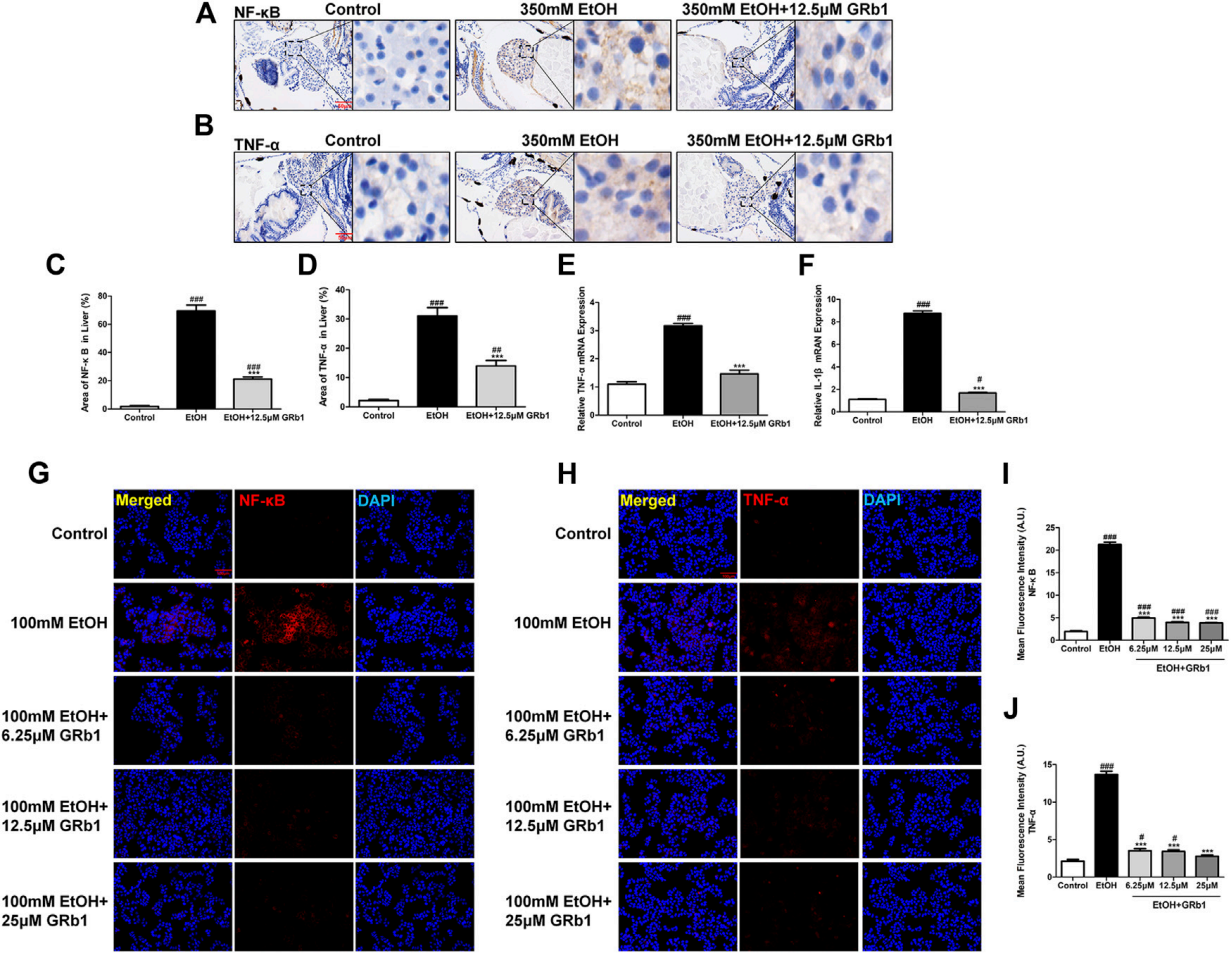
GRb1 downregulates the expression of proinflammatory cytokines. **(A)** NF-κB immunohistochemical staining of zebrafish larvae. Figures are magnified as 400ⅹ. **(B)** TNF-α immunohistochemical staining of zebrafish larvae. Figures are magnified as 400ⅹ. **(C)** Statistical analysis of the area of NF-κB expression in the liver of zebrafish larvae using ImageJ software (*n* = 6–8 per group). **(D)** Statistical analysis of the area of TNF-α expression in the liver of zebrafish larvae using ImageJ software (*n* = 6–8 per group). **(E)** and **(F)** Real-time PCR analysis of the mRNA levels of TNF-α and NF-κB in zebrafish larvae. The mRNA levels were normalized to β-actin mRNA levels and presented as fold change compared with the control group (*n* = 3 per group). **(G)** NF-κB immunofluorescence staining of L-O2 cells slides. Figures are magnified as 200ⅹ. **(H)** TNF-α immunofluorescence staining of L-O2 cell slides. Figures are magnified as 200ⅹ. **(I)** Quantitative analysis of NF-κB levels in L-O2 cells according to mean fluorescence intensity using ImageJ software. **(J)** Quantitative analysis of TNF-α levels in L-O2 cell according to mean fluorescence intensity using ImageJ software. Data are presented as the mean ± SEM. *p* < 0.05 (#), *p* < 0.01 (##), and *p* < 0.001 (###) compared with the control group; *p* < 0.05 (*), *p* < 0.01 (**), and *p* < 0.001 (***) compared with the 350 mM EtOH group or 100 mM EtOH group.

Nuclear factor-kappa B (NF-κB), an essential regulatory factor of inflammatory genes, immune cell proliferation, and oxidative stress (Janssen-Heininger et al., 2000), is required for the expression of several proinflammatory genes, including TNF-α and IL-1β (Liu et al., 2017). In zebrafish and L-O2 cells experiments, the alcohol-treated group showed increased NF-κB protein expression, while the GRb1 treatment groups expressed less NF-κB protein (Figures 8A,C,G,I). Subsequently, the level of the TNF-α cytokine increased after alcohol exposure but decreased after GRb1 treatment (Figures 8B,D,H,J). qPCR revealed that the mRNA expression levels of TNF-α and IL-1β were elevated in the ethanol exposure group but decreased after treatment with 12.5 μM GRb1 (Figures 8E,F). In conclusion, GRb1 reduced the expression of NF-κB and its downstream inflammatory factors, namely, TNF-α and IL-1β, thus reducing inflammatory damage in ALD.

## Discussion

The increasing incidence of ALD has become a significant public health burden. Because there are few treatments for ALD, the identification of new drugs effective for ALD is extremely urgent. A recent paper has shown that GRg1 protects hepatocytes against alcohol-induced injury by inhibiting inflammation, oxidative stress, and apoptosis (Li et al., 2018). GRb1, an abundant ginsenoside in ginseng, was demonstrated to play a protective role in acute ALD by inhibiting steatosis, oxidative stress, and inflammation in our study. Similar to GRg1, GRb1 also decreased the production of proinflammatory cytokines, such as TNF-α and IL-1β. Furthermore, our study provided preliminary information on the regulatory effect of GRb1 on neutrophil behavior in alcoholic liver injury. At the same time, both GRg1 and GRb1 have their own advantages in resisting alcohol injury. GRb1 acts distinctly on alleviating liver steatosis caused by alcohol, which can significantly alleviate lipid deposition in the liver and reduce triglyceride levels. However, GRb1 does not affect apoptosis like GRg1, for which no data were obtained in our study. Steatosis is the first stage of ALD, which is histologically defined as lipid accumulation in hepatocytes (Orman et al., 2013). In the present study, we observed apparent hepatic lipid deposition in alcohol-treated zebrafish larvae. This result was consistent with previous studies, in which 350 mM alcohol exposure causes hepatic lipid accumulation (Zhou et al., 2019). Next, we assessed the protective effect of GRb1 on acute alcohol-induced hepatic lipid deposition. Surprisingly, lipid droplets in the liver were significantly reduced after GRb1 administration. Correspondingly, the impact of GRb1 on lipid deposition was also verified in L-O2 cells.

However, alcoholic fatty liver can develop into alcoholic steatohepatitis with heavy alcohol intake. Alcoholic steatohepatitis is pathologically characterized by neutrophil infiltration and hepatocellular damage along with steatosis. Oxidative stress causes steatosis, lipid peroxidation, and inflammation, playing a crucial role in ALD (Li et al., 2015; Zhang et al., 2019). Studies have demonstrated that the metabolism of alcohol results in increased levels of cytochrome P450 family 2 subfamily E member 1 in hepatocytes and increased levels of nicotinamide adenine dinucleotide phosphate oxidase in hepatocytes, hepatic Kupffer cells, and infiltrated inflammatory cells, mainly contributing to the generation of ROS (De Minicis and Brenner, 2008; Abdelmegeed et al., 2017). GSH is a crucial factor in the endogenous protective system for eliminating ROS, and the lack of GSH in hepatocytes may disturb the antioxidant defense system, leading to ROS accumulation (Pan et al., 2020). In this study, we used DHE and NA-8 probes to visualize the level and distribution of ROS and GSH in the livers of live larvae. After stimulation by ethanol, ROS levels in the liver significantly increased, and GSH was heavily depleted. Dramatically, GRb1 reversed this condition, indicating that GRb1 reduced ROS accumulation and partially restored GSH, lowering MDA level and resisting oxidative damage.

Transmigration of neutrophils into the liver parenchyma has been considered a key driver of liver injury (Ramaiah and Jaeschke, 2007b) and to play a prominent role in ALD (Ramaiah and Jaeschke, 2007a). We used neutrophil-labeled zebrafish to intuitively and timely observe that GRb1 reduced neutrophil recruitment in the liver induced by alcohol. Of note, ROS from neutrophils have been deemed crucial in tissue damage (Ramaiah and Jaeschke, 2007a). Overall, GRb1 alleviates neutrophil infiltration in the liver during alcohol consumption, thus inhibiting possible further damage by the release of protease and oxidative stress.

NF-κB, an essential inflammatory transcription factor, is of great significance in regulating the signaling pathways related to pathological liver changes (Huang et al., 2019). Alcohol consumption increases gut permeability, causing translocation of bacterial products, such as lipopolysaccharide (Gao et al., 2019), and finally activating NF-κB to motivate the production of proinflammatory cytokines, such as TNF-α, IL-1β, and IL-6 (Neuman et al., 2015). In addition, oxidative stress enhances the activation of NF-κB via nicotinamide adenine dinucleotide phosphate oxidase, amplifying Kupffer cell production of TNF-α in turn (Kawaratani et al., 2013). TNF-α, an inflammatory cytokine, has been reported to be a hinge in alcoholic liver injury (Kitazawa et al., 2003), and the circulating level of TNF-α correlates with the severity of alcoholic hepatitis and alcoholic hepatitis mortality (Neuman et al., 2015). IL-1β is also a potent proinflammatory cytokine that is markedly increased in animal models and patients with ALD (Kawaratani et al., 2013). In this study, GRb1 was shown to lower the level of NF-κB increased by alcohol and reduce the expression of TNF-α and IL-1β. Decreased expression of these two effective inflammation-related factors and inhibition of neutrophil infiltration suggested that GRb1 prevents further inflammation damage to the liver caused by alcohol.

**FIGURE 9:**
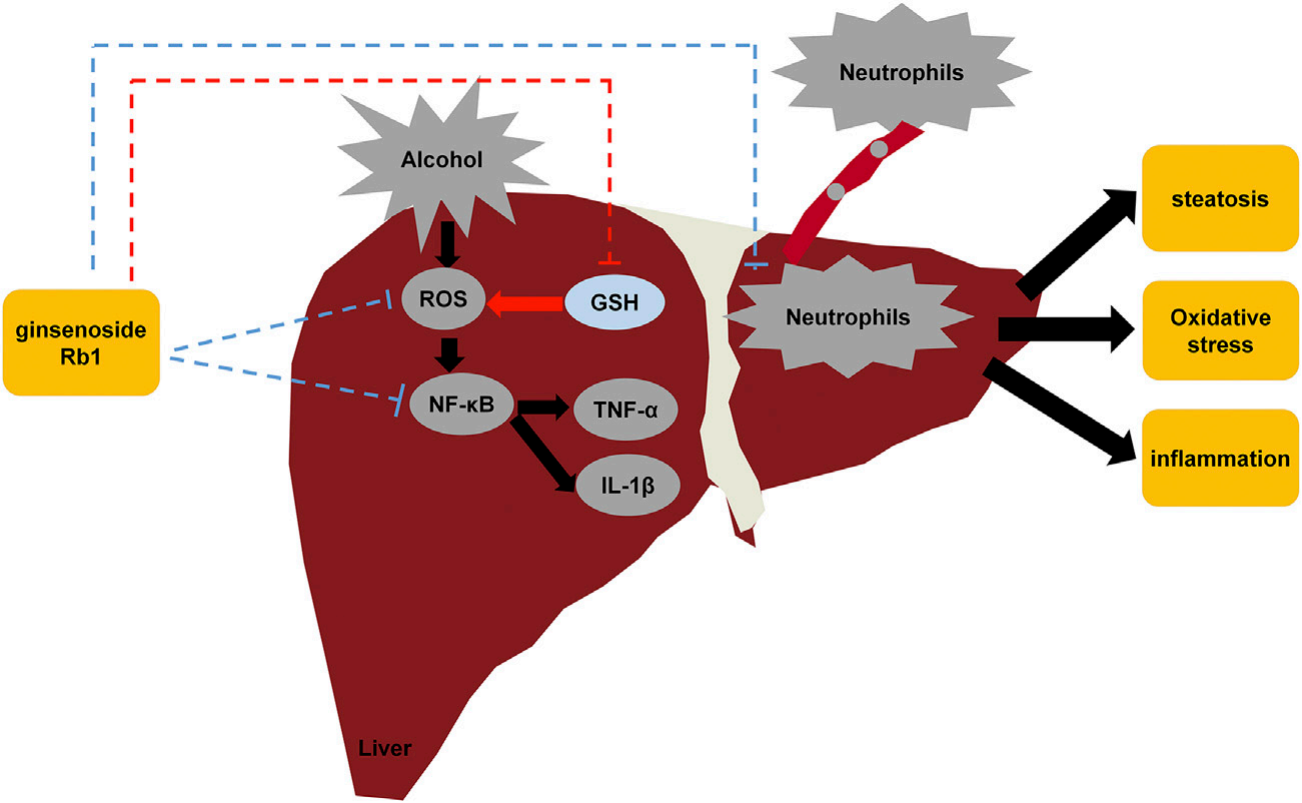
Diagram of the protective mechanisms of GRb1 against alcohol-induced liver injury.

Taken together, the results of our study suggest that GRb1 has a protective effect on ALD by lowering lipid accumulation, alleviating oxidative stress, and ameliorating inflammation induced by proinflammatory chemokines and neutrophil infiltration (Figure 9). However, further research is needed to elucidate how GRb1 regulates lipid metabolism and interferes with oxidative stress. In conclusion, our experimental study revealed the definite efficacy and possible mechanism of GRb1 in alcoholic liver injury treatment, providing scientific evidence for the clinical application of GRb1 against ALD.

## Data Availability

The original contributions presented in the study are included in the article/Supplementary Material; further inquiries can be directed to the corresponding authors.

## Abbreviations

ALD, alcoholic liver disease; DHE, dihydroethidium; DMEM, Dulbecco’s Modified Eagle Medium; EGFP, enhanced green fluorescent protein; EtOH, ethanol; GRb1, ginsenoside rb1; GSH, glutathione; H&E, hematoxylin and eosin; L-O2, human hepatocyte cell line; MDA, malondialdehyde; NF-κB, nuclear factor-kappa B; OCT, optimal cutting tissue; PBS, phosphate-buffered saline; ROS, reactive oxygen species; TNF-α, tumor necrosis factor α.
