# Supplementary material for: Ginsenoside Rb1 Alleviates Alcohol-Induced Liver Injury by Inhibiting Steatosis, Oxidative Stress, and Inflammation
Source: Front Pharmacol. 2021 Feb 26;12:616409. doi: 10.3389/fphar.2021.616409 (PMC7952325; doi:10.3389/fphar.2021.616409)
Supplement: Supplementary file 1 [file datasheet1.docx]

Supplementary Material

# Supplementary Data

## Toxicology of G-rb1 in zebrafish larvae

Morphological changes, survival rate, heart rate and body length were indexes to evaluate the toxicology of G-rb1 in zebrafish larvae comprehensively. Zebrafish larvae at 4dpf were treated with G-rb1 in a range of concentrations from 0μM to 200μM for 72 h. The tested concentrations of G-rb1 did not result in morphological abnormalities in zebrafish larvae (Fig. S1A), suggesting that G-rb1 did have no toxic effects on the developmental stages of zebrafish. Notably, the safety of G-rb1 was guaranteed to some extent, as even 200µM G-rb1 did not cause abnormal mass mortality in larvae (Fig. S1B). Compared with the control group, there was no inhibition or acceleration of heart rate in the drug group (Fig. S1C), suggesting that G-rb1 is no harm to cardiac function. We did not find any significant difference in body length between the control group and all the treatment groups (Fig. S1D). Based on the above toxicological results, we found that G-rb1 is safe to use within the concentration varying from 0μM to 200μM. Therefore, we selected 6.25μM, 12.5μM, and25μM G-rb1 for further experimentation.

# Supplementary Figure and Table

## Supplementary Figure 1

**
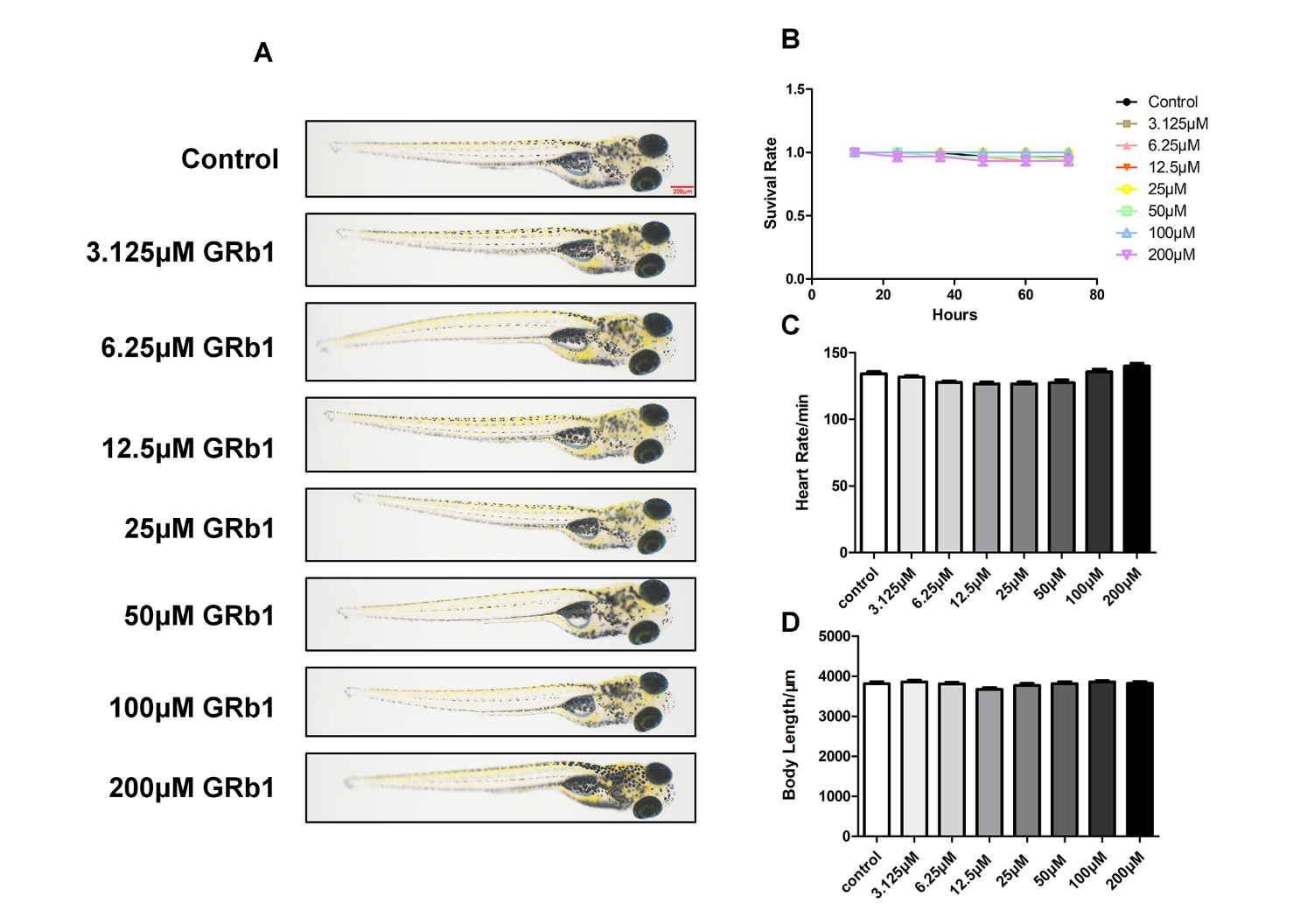
**

**Supplementary Figure 1.** (A) Assessment of developmental malformation. Figures are magnified as 20ⅹ. (B) survival rate. (C) Heart rate. (D) Body length. The data are displayed as the means ± SEM (n=10 per group). p<0.05 (#) vs. the control group.
